# Preferences for the selection of unique tRNA primers revealed from analysis of HIV-1 replication in peripheral blood mononuclear cells

**DOI:** 10.1186/1742-4690-2-21

**Published:** 2005-03-24

**Authors:** Kenda L Moore-Rigdon, Barry R Kosloff, Richard L Kirkman, Casey D Morrow

**Affiliations:** 1 Department of Microbiology, University of Alabama at Birmingham, Birmingham, Alabama 35294, USA; 2 Department of Cell Biology, University of Alabama at Birmingham, Birmingham, Alabama 35294, USA

## Abstract

**Background:**

All human immunodeficiency virus (HIV-1) uses a host tRNA^Lys,3 ^as the primer for reverse transcription. The tRNA^Lys,3 ^is bound to a region on the HIV-1 genome, the primer-binding site (PBS), that is complementary to the 18 terminal nucleotides of tRNA^Lys,3^. How HIV-1 selects the tRNA from the intracellular milieu is unresolved.

**Results:**

HIV-1 tRNA primer selection has been investigated using viruses in which the primer-binding site (PBS) and a sequence within U5 were altered so as to be complementary to tRNA^Met^, tRNA^Pro ^or tRNA^Ile^. Analysis of the replication of these viruses in human peripheral blood mononuclear cells (PBMC) revealed preferences for the selection of certain tRNAs. HIV-1 with the PBS altered to be complementary to tRNA^Met^, with and without the additional mutation in U5 to be complementary to the anticodon of tRNA^Met^, stably maintains the PBS complementary to tRNA^Met ^following extended *in vitro *culture in PBMC. In contrast, viruses with either the PBS or PBS and U5 mutated to be complementary to tRNA^Ile ^were unstable during *in vitro *replication in PBMC and reverted to utilize tRNA^Lys,3^. Viruses with the PBS altered to be complementary to tRNA^Pro ^replicated in PBMC but reverted to use tRNA^Lys,3^; viruses with mutations in both the U5 and PBS complementary to tRNA^Pro ^maintained this PBS, yet replicated poorly in PBMC.

**Conclusion:**

The results of these studies demonstrate that HIV-1 has preferences for selection of certain tRNAs for high-level replication in PBMC.

## Background

Although the major steps in reverse transcription have been known for some time, the process by which the tRNA primer is specifically selected from the intracellular milieu by the virus is less well understood. Even though different retroviruses select different tRNA primers for reverse transcription, within a group of retroviruses, the tRNA primer selected is conserved [1,2]. For example, murine leukemia virus (MuLV) selects tRNA^Pro^, while avian leukosis virus (ALV) selects tRNA^Trp ^[3,4]. Human immunodeficiency virus type 1 (HIV-1), as do all lentiviruses, selects tRNA^Lys,3 ^for use as the primer for reverse transcription [5,6]. How and why HIV-1 exclusively selects tRNA^Lys,3 ^as the primer for reverse transcription is not known. Pseudovirions composed of *Gag *and *Gag-pol *are enriched for tRNA^Lys^, including tRNA^Lys,3^, that is used for initiation of reverse transcription [2,7,8]. Additional studies have shown that the specific incorporation of lysyl tRNA synthetase and its interaction with *Gag *could also be important for the specific capture of tRNA^Lys,3 ^used for initiation of reverse transcription [9-11].

Substitution of the primer-binding site (PBS) to be complementary to alternative tRNAs results in the capacity of HIV-1 to transiently use these tRNAs for initiation of reverse transcription [12-14], even though upon extended culture, these viruses all reverted back to specifically utilize tRNA^Lys,3 ^for initiation of reverse transcription. In some instances, mutation of a region 5' of the PBS so as to be complementary to the anticodon of certain tRNAs, in conjunction with mutations of the PBS, results in the virus stably utilizing these alternative tRNAs for replication [15-19]. Interestingly, analysis of the virion tRNAs of a virus that stably utilized tRNA^His ^for replication did not show a difference in composition compared to that of the wild type virus that utilized tRNA^Lys,3^, indicating that tRNAs in the HIV-1 virion did not necessarily reflect the tRNA selected for initiation of reverse transcription [20].

The fact that HIV-1 can select different tRNAs for replication suggests the virus has access to a variety of tRNAs. Recent studies from this laboratory demonstrated that the tRNA selected by HIV-1 for replication have undergone nuclear-to-cytoplasmic transport. Furthermore, the tRNAs that are aminoacylated, indicating inclusion in translation, are most efficiently selected as primers [21]. The realization that tRNA biogenesis and translation might intersect with primer selection has prompted us to re-examine the stability and replication of HIV-1 with a PBS complementary to alternative tRNAs in a relevant cell type peripheral blood mononuclear cells (PBMC). In a previous study, we found that HIV-1 in which the PBS was altered to be complementary to tRNA^Lys1,2 ^or tRNA^His ^reverted to utilize tRNA^Lys,3 ^upon extended culture in PBMC [22]. Viruses could be generated which stably utilized these tRNAs for replication if additional mutations within the U5, consisting of nucleotides complementary to the anticodon regions, were also included in the viral genomes. Interestingly, viruses which utilize tRNA^Lys1,2 ^had further adapted to utilize this tRNA, exhibiting replication characteristics similar to the wild type virus following extended *in vitro *replication in human PBMC. Similar results have been recently reported for HIV-1 in which the PBS and a second region upstream, the primer activation site (PAS), has been altered to be complementary to tRNA^Lys1,2 ^[23]. In this case, the virus stably utilized tRNA^Lys1,2 ^for an extended culture period. A mutation in the RNase H domain of the reverse transcriptase was also found, although the major determinant of the stability of the PBS was correlated with the mutations in the PAS and PBS.

In the current study, we have further examined the preference of HIV-1 for certain tRNAs. A previous study from this laboratory has shown that viruses with a PBS complementary to tRNA^Pro ^or tRNA^Ile ^were unstable following replication in SupT1 cells, an immortalized, continuous, human T cell line [24]. However, during the process of reversion to a PBS complementary to tRNA^Lys,3^, we noted several different anomalies with the PBS, including the isolation of viruses with multiple PBS complementary to other tRNAs and a virus in which the PBS was complementary to tRNA^Met^. Further characterization of this virus revealed that it stably utilized tRNA^Met ^as the initiation primer following additional mutations in which the U5 was made complementary to the anticodon region of tRNA^Met ^[15,19]. We have now analyzed the replication and stability of the PBS of viruses in which the PBS alone was altered to be complementary to tRNA^Met^, tRNA^Pro ^or tRNA^Ile^, as well as viruses with both the PBS and U5 region altered to be complementary to the 3' 18-nucleotides and anticodon of these tRNAs. Clear differences were identified with respect to primer preference that correlated with virus replication. The results of these studies therefore establish that preferences for selection of certain tRNAs to be used in reverse transcription by HIV-1 do exist and are more evident following replication in PBMC than in continuous T cell lines.

## Results

### Construction and characterization of HIV-1 proviral genomes complementary to tRNA^Met^, tRNA^Pro ^and tRNA^Ile^

**Figure 1:**
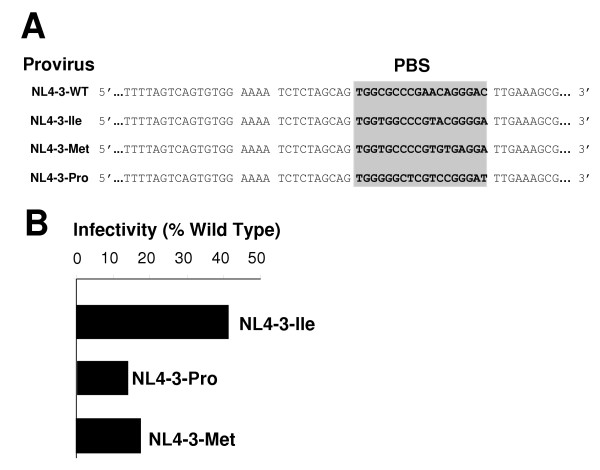
**U5-PBS sequence and infectivity levels of HIV-1 NL4-3 viral mutants at start of PBMC infection**. **Panel A**. HIV-1 U5 and PBS sequence shown (from 5' to 3'). Viral primer binding site (PBS) sequence was altered to be complementary to the 3' terminal 18 nucleotides of tRNA^Ile^, tRNA^Met ^and tRNA^Pro^. The PBS sequence is shadowed. **Panel B**. Comparison of infectivity of NL4-3 PBS mutants. HIV-1 NL4-3 proviral clones were transfected into 293T cells, incubated for 48 hours, and supernatants were measured for infectious units. For a given sample, the number of infectious units per microliter is equal to the number of blue cells in a well divided by the dilution factor for that well and represents the average of at least two wells. Wild type infectivity levels were set at 100% and mutant virus infectivity was reported as a percentage of wild type. All viruses with altered PBS sequences showed reduced levels of infectivity as compared to wild type. Results presented are representative of three experiments.

In previous studies, we have described the construction of HIV-1 proviral genomes in which the PBS was made complementary to alternative tRNAs [15-19]. For these studies, the proviral genomes were based on HXB2, which allows high-level replication in continuous T cell lines (eg. SupT1s). For the current studies, we have transferred the 5' LTR up to the *BssH*II site (nucleotide 233) from these clones into the NL4-3 proviral clone of HIV. The NL4-3 proviral clone of HIV-1, in contrast to the HXB clone, contains open reading frames for all the accessory proteins and replicates to high levels in PBMC. The U5-PBS regions of the subsequent proviral constructs, named pNL4-3-Met, pNL4-3-Pro and pNL4-3-Ile were sequenced prior to analysis to confirm that the constructs were isogenic with the wild type with the exception of the 3' 18-nucleotide PBS region (Figure 1A).

To characterize these viruses, we first measured the production of infectious virus and p24 antigen following transfection into 293T cells. Since 293T cells do not support HIV-1 replication, this analysis would provide us with the inherent infectivities of viruses prior to undergoing reverse transcription/replication in PBMC. In our previous studies, we noted that there was no substantial difference in the production of virus (as measured by p24 antigen) as a result of altering the PBS in the HXB2 proviral constructs [15-19]. For the current studies, we transfected the proviral clones into 293T cells and determined the amount of infectious units using the JC53BL assay; virus production was then measured using a p24 antigen capture ELISA. Infectivity was determined as the ratio of infectious units to p24 antigen. The values are presented relative to the infectivity of the wild type virus, with a PBS complementary to tRNA^Lys,3 ^(Figure 1B). All of the viruses with altered PBS had infectivities lower than the wild type. The virus NL4-3-Ile, with a PBS complementary to tRNA^Ile^, was consistently the most infectious of the mutants with a level approximately 40% that of wild type, while the other viruses were 10–20% as infectious as the wild type virus.

### Stability of PBS following replication in PBMC

**Figure 2:**
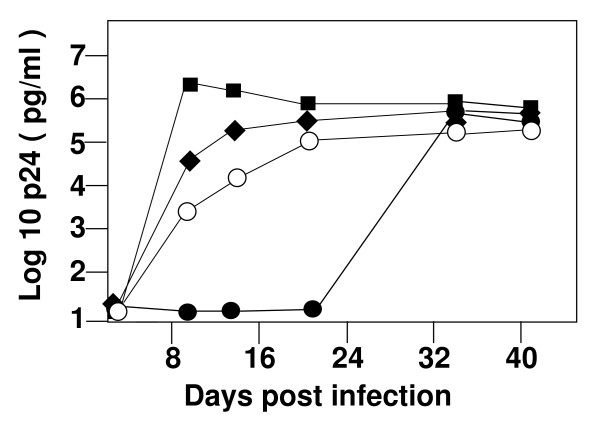
**Replication of HIV-1 with PBS sequence altered to be complementary to the 3' 18 nucleotides of tRNA^Ile^, tRNA^Met ^and tRNA^Pro ^in human peripheral blood mononuclear cells (PBMC)**. Infections were initiated with transfection supernatant containing approximately 200 pg of p24 antigen in a volume of 10 mLs of media, giving a final p24 level of 20 pg/mL on day zero. At 14 day intervals, 5 × 10^6 ^fresh PHA stimulated PBMC were added to each culture. Supernatants were assayed for p24 viral antigen using an ELISA. Two additional separate infections produced similar replication patterns for each virus. Squares are wild type NL4-3; diamonds are NL4-3-Met; open circles are NL4-3-Ile and closed circles are NL4-3-Pro.

We next wanted to determine the effects of alteration of the PBS on the replication of these viruses in PBMC. Infections were initiated with 200 pg of p24 and were allowed to proceed with re-feeding of PBMC every 14 days for periods of time exceeding 50 days of *in vitro *culture. The cultures were sampled periodically, supernatants were assayed for p24 antigen and cells were processed to extract high molecular weight DNA to determine the stability of the PBS. All of the viruses with an altered PBS showed an initial delay in production of p24 antigen compared to the wild type virus, consistent with the initial reduced infectivity compared to wild type (Figure 2). The NL4-3-Met virus had replication kinetics most similar to wild type virus in that during the first 10 days of culture we observed a rapid rise in p24 antigen, followed by a plateau at a level similar to that for wild type. The NL4-3-Ile virus replicated more slowly, with a gradual rise in p24 antigen before finally reaching a level similar to wild type. Finally, the NL4-3-Pro virus showed minimal replication in the first 21 days of culture, followed by a rapid increase in the next 14 days to reach levels similar to that of the wild type virus (Figure 2). Although all of the viruses replicated in PBMC, the kinetics did not correlate with the initial infectivities from the 293T transfection supernatants.

During the culture period, we collected the DNA from the cells to determine the status of the integrated virus PBS. The wild type virus, as expected, maintained a PBS complementary to tRNA^Lys,3 ^throughout the entire culture period (data not shown). In contrast, viruses with a PBS complementary to tRNA^Ile ^initially used tRNA^Ile ^for reverse transcription, but by day 21 from the initiation of culture had reverted to be complementary to tRNA^Lys,3 ^(Table 1). Viruses in which the PBS was altered to be complementary to tRNA^Pro ^(NL4-3-Pro) appeared to be slightly more stable and maintained the PBS complementary to this tRNA through day 21 of culture, before reverting to wild type by day 35. However, the subsequent rapid replication of the virus corresponded with the presence of a PBS complementary to tRNA^Lys,3 ^(Table 1).

**Table 1:** Stability of PBS following extended culture in PBMC

**Virus**	**PBS Sequence**	**Time to Reversion^1^(days)**
**NL4-3-lle**	Lys,3^**2**^	21
**NL4-3-Met**	Met^**3**^	---^**4**^
**>NL4-3-Pro**	Lys,3	35

1. PBS analyzed at the time of *in vitro *culture in PBMC and found to be wild type, complementary to tRNA^Lys,3^.

2. PBS complementary to tRNA^Lys,3^.

3. PBS complementary to tRNA^Met^.

4. Analysis of 34 TA clones of the PBS following 63 days in culture revealed all maintained a PBS complementary to tRNA^Met^.

Surprisingly, the viruses in which the PBS was complementary to tRNA^Met ^remained stable for use of tRNA^Met ^throughout the complete culture period (in this case, up to 63 days post initiation of culture). Analysis of 34 individual TA clones of the PBS from these viruses revealed that all were complementary to tRNA^Met ^(Table 1). This is the first instance in which we have found a virus that stably maintains a PBS complementary to an alternative tRNA (not tRNA^Lys,3^) following extensive *in vitro *replication that did not have additional mutations in U5. Previously, analysis of this virus in the HXB2 proviral clone revealed that the PBS was unstable following replication of the virus *in vitro *in SupT1 cells and reverted back to use tRNA^Lys,3 ^[15,18]. We further characterized the replication of this virus compared to wild type virus at different times during the culture period. Analysis of p24 antigen production from this virus at day 56 post initiation of culture revealed that it replicated similar to the wild type virus, albeit with slightly lower levels of p24 antigen (data not shown). The infectivity of the virus obtained after long-term culture, which utilized tRNA^Met^, was approximately 50–80% of the wild type virus (data not shown). Collectively, the results of these studies establish that HIV-1 has a preference for certain tRNAs, such as tRNA^Met^, for the selection as primer for reverse transcription.

### Effect of mutations in U5 on replication of viruses that use alternative tRNAs

**Figure 3:**
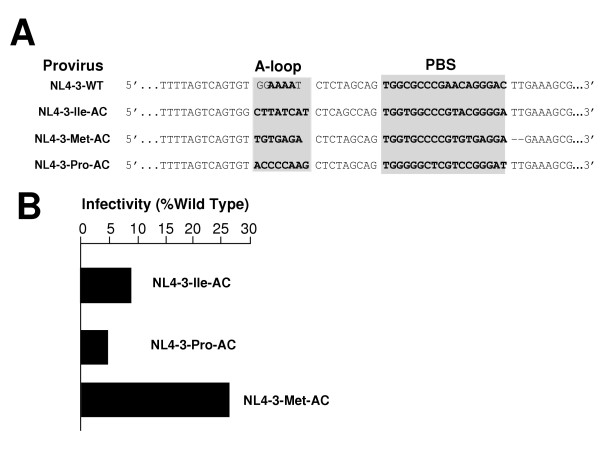
**U5-PBS sequence and Infectivity levels of HIV-1 NL4-3 viral mutants with altered U5 and PBS sequences at start of PBMC infection**. **Panel A**. HIV-1 U5 and PBS sequence (shown 5' to 3'). Viral primer binding site (PBS) and U5 A-loop sequences were altered to be complementary to the 3' terminal 18 nucleotides and anticodon loop of tRNA^Ile^, tRNA^Met^, tRNA^Pro^, and tRNA^Trp^, respectively. U5 A-loop sequence and PBS are shadowed. **Panel B**. Comparison of the infectivity of U5-PBS mutant NL4-3 viruses. HIV-1 NL4-3 proviral clones were transfected into 293T cells, incubated for 48 hours, and supernatants were measured for infectious units. For a given sample, the number of infectious units per microliter is equal to the number of blue cells in a well divided by the dilution factor for that well and represents the average of at least two wells. Wild type infectivity levels were set at 100%, and mutant virus infectivity was reported as a percentage of wild type. All viruses with altered U5 and PBS sequences had reduced levels of infectivity as compared to wild type virus. The data presented are representative for three independent experiments.

**Figure 4:**
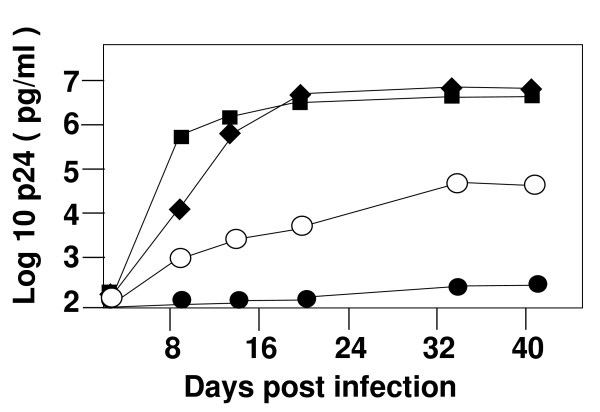
**Replication of HIV-1 with U5 and PBS sequence altered to be complementary to the anticodon loop and 3' 18 nucleotides of tRNA^Ile^, tRNA^Met^, tRNA^Pro ^and tRNA^Trp ^in PBMC**. Infections were initiated with transfection supernatant containing approximately 200 pg of p24 antigen in a volume of 10 mLs of media, giving a final p24 level of 20 pg/mL on day zero. Supernatants were assayed for p24 viral antigen every 7 days for a period of 42 days. At 14-day intervals, 5 × 10^6 ^fresh PHA stimulated PBMC were added to each culture. Two additional separate infections produced very similar replication patterns for each virus (data not shown). Squares are NL4-3 wild type; diamonds are NL4-3-Met-AC; open circles are NL4-3-Ile-AC; closed circles are NL4-3-Pro-AC.

In previous studies, we have found that with a PBS complementary to tRNA^Lys1,2^, tRNA^Met^, tRNA^Glu ^and tRNA^His^, the additional mutation in which the U5 region was made complementary to the anticodon stabilized the HXB2 proviral clones to allow continuous use of the alternative tRNA during replication [16,19,24,25]. In contrast, in the HXB2 provirus, modification of the U5 region for viruses in which the PBS was made complementary to tRNA^Pro ^or tRNA^Ile ^did not result in virus that could stably utilize these tRNAs following replication [24]. To determine if this would be case for viruses that were grown in PBMC, we constructed HIV-1 in which both the U5 and PBS were made complementary to tRNA^Met^, tRNA^Pro ^or tRNA^Ile ^(Figure 3A). The initial infectivities of the viruses were analyzed following transfection of the proviral clones into 293T cells. Similar to what we observed for viruses with just the PBS altered to be complementary to these tRNAs, the viruses with both the U5 and PBS altered demonstrated infectivities lower than wild type virus. In this case, the levels ranged from a low of 5% (NL4-3-Pro-AC) to a high of 30% (NL4-3-Met-AC) (Figure 3B). We initiated infections in PBMC with the same amount of p24 antigen. We noted a delay in the production of p24 antigen in the cultures of viruses in which both the PBS and A loop were mutated to be complementary to the alternative tRNA^Met ^or tRNA^Ile^, relative to the wild type virus (Figure 4). By day 21, the viruses derived from pNL4-3-Met-AC had p24 antigen levels in the culture supernatants similar to that for the wild type virus. Viruses derived from pNL4-3-Ile-AC replicated at levels approximately 1/10 that of the wild type virus, while viruses derived from pNL4-3-Pro-AC did not replicate well (or at all), as evidenced by p24 levels that did not increase substantially over the culture period (Figure 4).

We next analyzed the PBS of the viruses. Consistent with our previous studies, we found that the viruses in which both the U5 and PBS were complementary to tRNA^Met ^remained stable during the culture period (Table 2). Sequence analysis of the virus that stably utilized tRNA^Met ^(NL4-3-Met-AC) revealed a few nucleotide changes outside of the PBS. Previous studies from our laboratory have reported single nucleotide changes, noting that these changes might be important in stabilizing RNA structures to facilitate more effective primer selection [15,17-20,24]. Further experiments will be needed to address this issue. Characterization of NL4-3-Met-AC after extended culture revealed that it had infectivities that were still lower than that of the wild type virus (data not shown). In fact, the infectivities of the virus derived from pNL4-3-Met-AC were generally lower than those from the virus derived from pNL4-3-Met (data not shown). In contrast, viruses in which the PBS and U5 region were made complementary to tRNA^Ile ^were not stable and reverted to utilize tRNA^Lys,3 ^following *in vitro *replication (Table 2). Thus, the A loop modification did not stabilize the virus to continuously use tRNA^Ile ^for replication in PBMC. Virus with both the U5 and PBS altered to be complementary to tRNA^Pro ^replicated poorly in the *in vitro *culture. Amplification of the region containing the PBS required use of a double PCR method in which the initial PCR product was used as the template of the second reaction (double PCR). Sequence analysis revealed that NL4-3-Pro-AC had maintained a PBS complementary to tRNA^Pro ^(data not shown). Viruses in which the U5 and PBS were made complementary to tRNA^Pro ^also reverted to use wild type following replication in SupT1 cells; in this case, we found viruses which contained multiple PBS, some of which were complementary to tRNA^Lys,3^. Following replication in PBMC, though, we did not isolate viruses with multiple PBS and all the viruses isolated contained a PBS complementary to tRNA^Pro^. Collectively, the results of these studies establish that HIV-1 does have a preference for tRNA^Met ^over tRNA^Pro ^with respect to the selection of the tRNA primer for replication. Furthermore, tRNA^Ile ^is not favored for selection by HIV-1 even if compensatory mutations are provided in which the U5 region has been made complementary to the anticodon region.

**Table 2:** Analysis of U5-PBS from viruses following extended *in vitro *culture in PBMC

**Virus**	**U5-PBS**		**Days Post-Culture**
	Ile^3^		
**NL4-3-Ile-AC**	^1^5' AGTCAGTGT**TTATCAG**CTCTAGCAG ^2^**TGGTGGCCCGTACGGGGA **TTGAAA 3'	Input^5^	0
	Ile		
	^4^5' ************************* ****************** ****** 3'	PCR Product^6^	21
	Lys, 3		
	5' ************************* **TGGCGCCCGAACAGGGAC **-***** 3'	6/7 TA Clones^7^	35
	Lys,1,2		
	5' ************************* **TGGCGCCCAACGTGGGGC **-***** 3'	1/7 TA Clones	35
	Lys, 3		
	5' ************************* **TGGCGCCCGAACAGGGAC **-***** 3'	PCR Product	73
			
	Met		
**NL4-3-Met-AC 5**	5' AGTCAGTGTT**GTGAGA**CTGTAGCAG **TGGTGCCCCGTGTGAGGC **GAAAGC 3'	Input	0
	Met		
	5' ************************* ****************** ****** 3'	5/10 TA Clones	35
	5' ************************* *****************A ****** 3'	3/10 TA Clones	35
	5' ********************* *A* * ****************** ****** 3'	1/10 TA Clones	35
	5' ************************* *******T********** ****** 3'	1/10 TA Clones	35
	Met		
	5' ************************* ****************** A***** 3'	6/9 TA Clones	63
	5' ************************* *****************A A***** 3'	2/9 TA CIones	63
	5' *********C*****G********* ****************** A***** 3'	1/9 TA CIones	63

1. The U5-loop is in bold type.

2. Spaces separate the PBS (indicated in bold type) from flanking sequence.

3. PBS complementary to the 3' terminal 18-nucleotide sequence of the indicated host tRNA.

4. Asterisks represent conserved nucleotides.

5. "Input" refers to the clone that was used to initiate viral infection in PBMCs.

6. PCR product that was sequenced directly.

7. Refers to TA clones of PCR product that is cloned into the Promega, P-Gem T-Easy Vector System I, to isolate individual colonies for sequencing.

## Discussion

In previous studies, we have described HIV-1 in which the PBS and U5 have been altered to be complementary to tRNA^Met^, tRNA^Pro ^and tRNA^Ile ^[18,24]. All of the viruses with only a PBS complementary to these tRNAs were replication competent but reverted to the wild type following infection in SupT1. To extend these studies to a more relevant cell type, we cloned the mutant PBS into the NL4-3 background, which replicates well in PBMC, reaching high levels of p24 antigen in the culture supernatant. Analysis of the effect of altering the PBS on infectivity of proviral clones revealed that these viruses were 10–40% as infectious as the wild type virus, with the virus containing a PBS complementary to tRNA^Ile ^being the most infectious. However, analysis of the growth of these viruses revealed a clear preference for the viruses with a PBS complementary to tRNA^Met ^compared to the virus with a PBS complementary to tRNA^Ile^. Virus with a PBS complementary to tRNA^Pro ^had a rapid increase in p24 antigen after 21 days in culture and subsequently replicated similar to wild type and viruses with a PBS complementary to tRNA^Met^. As we had found in our previous studies, the sequence analysis of the PBS from both of these viruses at different times of culture revealed the reversion of the PBS to wild type [24]. The unexpected result from our studies was the distinct preference for HIV-1 to utilize tRNA^Met ^as evidenced by the stability of the PBS following long-term culture. The preference of HIV-1 for the selection of tRNA^Met ^was noted in a previous study in which we found a PBS complementary to this tRNA following analysis of the reversion of viruses that initially had a PBS complementary to tRNA^Trp ^[24]. A subsequent study found that HXB2 derived viruses in which only the PBS was mutated to be complementary to tRNA^Met ^reverted back to the wild type PBS; a virus that could stably use tRNA^Met ^was obtained by additional mutations in the U5 [15]. Thus, the results of our current study are unique in that the NL4-3-Met, without mutations in the U5, was stable and replicated well in PBMC, at a level comparable to the wild type virus. Further characterization of the viruses obtained from these two cell types will be needed to resolve the reason for differences in stability of the PBS. It is possible that differences in nucleotide concentrations or tRNA availability between the SupT1 or PBMC could influence the stability of the PBS. Further experiments using an endogenous RT reaction [18] and analysis of virus tRNA^Met ^content could be informative. With respect to the latter point though, our previous studies have not shown differences in tRNA content of virions that use alternative primers for reverse transcription [20].

How does this relate to the process of primer selection? In recent studies, we have found that HIV-1 most effectively selects tRNAs that have undergone the steps in tRNA biogenesis that result in transport from the nucleus to the cytoplasm [21]. Once in the cytoplasm, the tRNAs interact with a myriad of proteins involved in translation [26]. At any one time, the tRNA selected by HIV-1 as a primer for reverse transcription has been channeled into the translational process, supporting the idea of coupling of translation and primer selection. One possibility could be the coupling of primer selection with the synthesis of the *Gag-pol *polyprotein. Previous studies have shown that pseudovirions composed of *Gag *and *Gag-pol *contain the appropriate ratios of tRNA^Lys ^found in intact wild type virions [7,8]. That is, during the translation of *Gag-pol*, the tRNAs available for selection might be enriched for tRNA^Lys,3 ^and tRNA^Met^; conversely, tRNA^Ile ^may not be favored because of the absence of isoleucine during translation of *Gag-pol*. This is not because isoleucine is excluded from the *Gag-pol *protein. Rather, it is possible that a translational event in the production of *Gag-pol*, possibly at or during the frame shifting, could influence the local amounts of tRNA so as to favor some (e.g., tRNA^Lys,3^, tRNA^Met^) while not others (e.g., tRNA^Ile^). Without tRNA^Ile ^to occupy the PBS, there would be greater access by tRNA^Lys,3 ^to facilitate reversion back to the wild type PBS, complementary to tRNA^Lys,3^. Viruses with a PBS complementary to tRNA^Pro^, and from previous studies those with PBS complementary to tRNA^His^, tRNA^Lys1,2 ^or tRNA^Glu^, initially replicated slowly but reverted to use tRNA^Lys,3^, whereupon they exhibited rapid replication. We would predict that the local availability of these tRNAs would be sufficient to allow the limited replication. However, given the selective pressure for the use of tRNA^Lys,3^, the virus would have a propensity to revert to wild type if the tRNAs were not present at levels similar to tRNA^Lys,3 ^or tRNA^Met^. Coupling of the synthesis of *Gag-pol *with primer tRNA selection and encapsidation might provide all of the necessary components for the generation of infectious virus within the same intracellular locale. Further studies will be needed to explore the relationship between the synthesis of *Gag-pol *and primer selection using the unique viruses described in this study.

The results of our studies in which we included additional regions of complementarity between the tRNA and U5 further substantiates a role for this interaction in the selection of the tRNA primer [24]. In a recent study, we found that viruses with PBS and U5 complementary to tRNA^Lys1,2 ^or tRNA^His ^were stable after extended replication in PBMC, similar to what we found for NL4-3-Met-AC [22]. In this study, the virus derived from NL4-3-Pro-AC replicated poorly and in contrast to NL4-3-Pro, did not revert to wild type following extensive *in vitro *culture in PBMC. This finding supports the idea that the complementarity between the U5 and tRNA can impact the selection process. Most probably, the NL4-3-Pro-AC remains stable because it can more effectively select tRNA^Pro^, or exclude tRNA^Lys,3^, from binding to the PBS complementary to tRNA^Pro^. If tRNA^Lys,3 ^is used, the PBS generated during plus strand synthesis would be complementary to tRNA^Lys,3^, which could facilitate reversion upon subsequent replication. The results of the current study and others are consistent with the concept that multiple interactions between the viral RNA genome and tRNA occur during the selection process [16,19,23,24]. A recent study found that a virus that stably used tRNA^Lys1,2 ^could be generated by changing the PBS and a region upstream, different from the A loop, designated as the primer activation site (PAS) [23]. Interestingly, a virus with similar mutations to facilitate the use of tRNA^Pro ^was not stable, consistent with the results presented in our study. We suspect that the U5-PBS interactions are more important for tRNA selection in primary cells (e.g., PBMC) where the availability of the tRNAs in the intracellular environment might be different. Further experiments will be needed to address this issue.

In summary, the results of our studies analyzing the replication in PBMC of HIV-1 with PBS complementary to alternative tRNAs has revealed a clear preference for certain tRNAs to be selected for replication. The tRNA^Met ^is highly favored for selection, slightly less than the wild type tRNA^Lys,3^, while tRNA^Ile ^is not favored for selection as evidenced by the fact that viruses with this U5-PBS revert to use tRNA^Lys,3 ^after short term culture. Viruses that use tRNAs such as tRNA^Pro^, tRNA^Lys1,2 ^or tRNA^His ^replicate poorly in PBMC compared to the wild type virus and the virus that uses tRNA^Met ^[22]. These results suggest that HIV-1 can select the tRNA primer from a pool of tRNAs, with certain tRNAs favored over others, further substantiating a link between viral protein translation and primer selection.

## Conclusion

The results of our study provide new insights into the tRNA selection process by HIV-1. For the first time, we have described a unique HIV-1 that utilizes a tRNA primer (tRNA^Met^) that does not require additional mutations within the U5. This virus replicates well in human PBMC, similar to the wild type virus. In contrast, the virus did not prefer to select tRNA^Ile ^as evidenced by the fact that this virus was unstable with or without additional mutations within U5. This result highlights that different tRNAs are available in PBMC for capture by HIV-1 for use as the primer for reverse transcription. The importance of additional mutations within U5 that are complementary to the anti-codon region of tRNAs for selection was highlighted by the studies with viruses in which the PBS was made complementary to tRNA^Pro^. In this case, the virus was unstable with only the PBS complementary to tRNA^Pro ^while the additional U5 mutation did not allow reversion but severely impacted on the subsequent replication capacity of the virus, demonstrating that complex RNA-RNA interactions occur within the U5-PBS during primer selection. Collectively, the results of our studies demonstrate, for the first time, that distinct preferences exist for the selection of tRNAs to be used as the primer for HIV-1 reverse transcription. Coupled with our previous studies, we conclude there is most probably a link between viral translation and primer selection. The exclusive use of tRNA^Lys,3 ^by HIV-1 is most probably due to inherent features of this tRNA as well as the intracellular availability during viral translation.

## Methods

### Construction of NL4-3 proviruses containing modified PBS regions

We previously reported the construction of pHXB2 (Met and Met-AC) and pHXB2 (Ile and Ile-AC) and PHXB2 (Pro and Pro-AC) with PBS and PBS-U5 changes complimentary to the respective tRNA 3' and anticodon sequences [15,18,19,24]. These proviral mutants were constructed in the pHXB2 molecular clone of HIV-1. In this study, the NL4-3 molecular clone of HIV-1 was used as the proviral backbone DNA for the U5-PBS mutants [27]. Proviral clones pHXB2 (Met, Met-AC, Pro, Pro-AC, Ile and Ile-AC) from these previous studies were digested with *Hpa*I and *BssH*II restriction enzymes (New England Biolabs, Beverly, MA) to release an 868-bp fragment that contained the 5' LTR, PBS, and leader region from the *gag *gene of HXB2. The *Hpa*I site is located upstream of the 5' LTR within the flanking sequence, and the *BssH*II site is located downstream of the PBS within the viral genome, in the proximity of nucleotide 255 (5'GCGCGC-3'). Digests were run on a 1% Agarose gel (Amresco, Solon, OH) to separate the 868 bp U5-PBS fragment from the pHXB2 proviral DNA fragment. U5-PBS fragments were isolated using the Qiagen Gel Extraction kit (Qiagen, Valencia, CA) and cloned into the NL4-3 HIV-1 proviral plasmid using the same *Bss*HII and *Hpa*I restriction sites. All resulting NL4-3 constructs were verified by DNA sequencing to ensure the identity of the mutated sequence and the successful ligation of the U5-PBS fragment into the pNL4-3.

### Tissue Culture and DNA transfections

Transfections were performed according to the protocol for the Fugene 6 Transfection Reagent (Roche Molecular Biochemicals, Indianapolis, IN). Briefly, 2 μg of proviral plasmid DNA and 3 μL Fugene reagent were added to 100 μL of Dulbecco's modified Eagle's Medium (no Fetal Bovine Serum) (Cellgro by Mediatech, Herndon, VA). This mixture was incubated at room temperature for approximately 45 minutes then added drop-wise to one well of a 6-well plate containing 60% confluent 293T cells in DMEM with 10% Fetal Bovine Serum (FBS). The transfections were incubated overnight at 37°C and the medium was replaced with fresh DMEM containing 10% FBS (Hyclone, Logan, UT). After 48-hours, all supernatants were collected and stored at -80°C. Supernatants from transfected cells were assayed for HIV-1 p24 antigen (Beckman Coulter, Miami, FL) and infectivity [28].

### PBMC Infections

Human peripheral blood mononuclear cells (PBMC) were collected, stimulated using rIL-2 phytohemagglutinin (PHA) (Sigma, St. Louis, MO) and maintained as described previously [22]. Infections were performed by innoculating 20 × 10^6 ^PHA-stimulated PBMC with a volume of transfection supernatant containing 200 pg of p24 antigen and incubating for 2 hours at 37°C and 5% CO_2_. Virus/PBMC mixtures were transferred to 25 cm^2 ^tissue culture flasks, and the final volumes were adjusted to 10 mL with RPMI 1640, 1× (Cellgro by Mediatech, Herndon, VA) containing 15% FBS (Hyclone, Logan, UT) and 30 U/mL rIL-2 (Sigma, St. Louis, MO).

Infected PBMC cultures were maintained for 10 weeks by replacing half the volume of medium every 7 days, without removing PBMC. Every 7 days, 1 mL of cell suspension was removed and centrifuged in an Eppendorf microcentrifuge at 24,000 × *g *for 2 minutes. Supernatant was separated from the cell pellet and stored at -80°C for further analysis by p24 ELISA and JC53BL infectivity assays. Cell pellets were also stored at -80°C for isolation of high molecular weight DNA. Every 14 days an additional 5 × 10^6 ^PHA-stimulated PBMC were added to each culture.

### Infectivity Assay

Levels of infectious virus (IU/μL) in both 293T and PBMC culture supernatants were determined using the JC53BL assay as previously described [22,28]. For a given test sample, the number of infectious units per microliter is equal to the number of blue cells in a well divided by the dilution factor for that well and represents the average of at least two wells.

### PCR analysis of integrated PBS-containing proviral DNA

Cell pellets from virus cultures were stored at -80°C, and isolation of high molecular weight genomic DNA was performed as described previously [22]. Approximately 2 μg of each genomic DNA sample was PCR amplified using 5 pmole/μL *EcoRI *(5'-CGGAATTCTCTCCTTCTAGCCTCCGCTAGTC-3') and 5 pmole/μL *SphI *(5'-CCTTGAGCATGCGATCTACCACACACAAGGC-3') primers (Gibco BRL, Rockville, MD) with 2.5 mM dNTP, 50 mM MgC1_2 _(Invitrogen, Grand Island, NY), and 5 U/μL Recombinant TAQ DNA polymerase (Invitrogen, Grand Island, NY). The PCR program used to amplify genomic DNA had a denaturation temperature of 94°C and an annealing temperature of 56°C. The resulting PCR product was isolated and purified as described previously [22]. In cases of low virus replication (eg. pNL4-3-Pro-AC), the PCR product was used as a template for an additional PCR reaction (referred to as double PCR).

### Subcloning of PCR products and DNA Sequencing

Purified PCR product was sequenced for the U5-PBS region of the viral genome using the *EcoRI *primer (Invitrogen, Grand Island, NY). DNA sequencing was performed on an automated DNA sequencer. PCR products that resulted in accordant sequence throughout the U5-PBS region were considered to be a homogenous infection of virus, using the same tRNA primer. PCR products that resulted in discordant sequence in the PBS region were considered to be a heterogeneous population of virus, using more than one tRNA to prime reverse transcription, and was therefore, subjected to further TA cloning in order to isolate sequences of individual viruses. This PCR product was subcloned according to the Promega pGEM-T Easy Vector System I (Promega, Madison, WI) to prepare the DNA for efficient and accurate sequencing. PCR product was ligated into the pGEM-T Easy plasmid vector at 4°C overnight. Ligations were then transformed into DH5α *Escherichia coli *cells (Invitrogen, Grand Island, NY) and grown overnight on LB with 100 μg/mL ampicillin and 20 mg/mL Xgal. White colonies (indicating successful ligation) were picked and grown in LB-Amp100 μg/mL broth overnight at 37°C. DNA was harvested using the Qiagen QIAprep Spin miniprep kit, according to protocol (Qiagen, Valencia, CA). To assure that the TA clone DNA contained the PCR product insert, samples were digested by *EcoRI *to release the ligated fragment. Digests were run on a 1% agarose gel to verify the presence of a band of approximately 750 bp size. The TA clone DNA was then sequenced for the U5-PBS region using the *EcoRI *primer.
